# Bio-coloration of bacterial cellulose assisted by immobilized laccase

**DOI:** 10.1186/s13568-018-0552-0

**Published:** 2018-02-13

**Authors:** Ji Eun Song, Jing Su, Jennifer Noro, Artur Cavaco-Paulo, Carla Silva, Hye Rim Kim

**Affiliations:** 1 Department of Clothing and Textiles, Sookmyung Women’s University, Cheongpa-ro-47-gil 100 (Cheongpa-dong 2ga), Yongsan-gu, Seoul, 04310 South Korea; 2 Centre of Biological Engineering, University of Minho, Campus of Gualtar, 4710-057 Braga, Portugal; 3 Key Laboratory of Eco-Textile Ministry of Education, Jiangnan University, Wuxi, China

**Keywords:** Bacterial cellulose, Laccase, Polymerization, Bio-coloration

## Abstract

In this work a process for the bio-coloration of bacterial cellulose (BC) membranes was developed. Laccase from *Myceliophthora thermophila* was immobilized onto BC membranes and retained up to 88% of residual activity after immobilization. Four compounds belonging to the flavonoids family were chosen to test the in situ polymerase activity of immobilized laccase. All the flavonoids were successfully polymerized by laccase giving rise to yellow, orange and dark brown oligomers which conferred color to the BC support. The optimal bio-coloration conditions were studied for two of the tested flavonoids, catechol and catechin, by varying the concentration and time of incubation. High color depth and resistance to washing were obtained for both compounds. The highly porous bacterial cellulose material demonstrated great performance as a bio-coloration support, in contrast to other materials cited in literature, like cotton or wool. The process developed is presented as an environmentally friendly alternative for bacterial cellulose bio-coloration and will contribute deeply for the development of new fashionable products within this material.

## Introduction

The new challenges faced by the textile industry have lead to the development of new materials produced by environmentally friendly methodologies. The conventional dyeing of textiles is dependent on their chemical structure and requires the use of a wide range of dyes and auxiliaries, aggressive pH and elevated temperatures (Calafell et al. 2007). The polymers, synthesized from natural materials in the presence of oxidoreductases, have been explored as potential dyestuffs for textile coloration (Bai et al. 2016; Calafell et al. 2007; Kim et al. 2008). The application of enzymes in the textile dyeing/coloration provides therefore alternative possibilities of processing conditions such as milder temperature, pressure and pH conditions, and water savings (Su et al. 2017). Laccases (benzenediol:oxygen oxidoreductase, EC 1.10.3.2) as multi-copper-containing oxidoreductases, can efficiently catalyse the polymerization of phenolics. They are suitable tools for the oxidation of small colorless aromatic compounds, such as amines and phenols to aryloxy-radicals, which may undergo further non-enzymatic reactions resulting in colored dimeric, oligomeric and polymeric products (Morozova et al. 2007). For this reason, there has been increasing the interest in the application of laccases for the coloration of textiles by means of the formation of colored compounds. The products of enzymatic polymerization of some substrates may undergo colouration from yellow to orange to dark brown and impart colour to fibres when applied in situ. This catalyst has been applied for the enzymatic colouration of several textile fibres like wool, cotton or flax (Mikolasch and Schauer 2009). Shin et al. successfully tried the enzymatic assisted-dyeing of wool using several phenolic compounds namely catechol, guaiacol, dopamine, hydroquinone and ferulic acid as HRP or laccase substrates (Shin et al. 2001). Bai et al. described a new type of coloration system for wool fabrics using laccase. The results showed that the dyeing effect of the wool fabric samples using the single step processing method of in situ color synthesis and fabric dyeing was better than those dyed using the two-step methods of color synthesis and fabric dyeing under the same conditions. The color depth of the dyed wool fabrics increased gradually with increasing concentration of laccase, and also depended on other process parameters, such as dosage of catechol, temperature, and pH (Bai et al. 2016). Non-toxic and non-harmful phenolic substrates such as flavonoids (rutin, morin and quercetin) have been used to coat and colourise cotton and flax fabrics. The laccase-oxidised flavonoids can graft onto the surface of the cotton and provide a yellow to brown colour of different colour strength, depending on the type of external flavonoids used and the reaction conditions (Kim et al. 2007). A new process for the coloration of flax fabrics via enzymatic oxidation of natural flavonoids (morin, quercetin) has also been developed by Kim and co-workers (Kim et al. 2008). Laccase from *Trametes hirsuta* was able to react with flavonoids and polymerize them, resulting in a strongly colored polymeric solution which can be applied for the coloration of flax fabrics.

Bacterial cellulose (BC) is being used as a raw material for the development of many products with application in the textile area. This bio textile looks like as a gelatinous pulp film formed by interlacing cluttered of microfibrils, when being developed from the cultivation of microorganisms in an aerobic aqueous medium. These micro association of bacteria and yeasts, forms a kind of fungus that accumulates between the surface of the liquid and the liquid portion. The membrane of BC, when drought, presents a considerable tensile strength and durability, as well as natural dyeing accessibility. It is also able to be assembled as a garment by the traditional processes of pattern making and sewing (Ashjaran et al. 2013) creating new opportunities in a specific market niche. Due to its unique properties, several authors have been exploring the possibility to spin fibers obtaining material with acceptable tensile strength (Gao et al. 2011; Guhados et al. 2005).

This material has been used as support for the laccase immobilization however without clear description of the practical applications (Chen et al. 2015; Sampaio et al. 2016; Wu et al. 2017). We have previously optimized the swelling of bacterial cellulose membranes obtained from Scooby, in order to achieve the highest level of molecules entrapment and enzyme immobilization. The highly porous material showed potentialities for the in situ phenolics polymerization (Song et al. 2017).

Herein, our goal is to take advantage of the ability of phenolics to be polymerized by laccase, to biologically colorize the surface of bacterial cellulose membranes. A first study was conducted involving the immobilization of the enzyme onto BC and the in situ polymerization of different phenolics, namely catechol, catehin, hydroquinone and ferulic acid (Table 1). The new oligomers formed by enzymatic action were characterized by ^1^H NMR and MALDI-TOF spectrometry. The best conditions of BC biocoloration were then optimized for two flavonoids, catechol and catechin. The color depth and resistance were evaluated spectrophotometrically.

**Table 1:** Flavonoid compounds used for enzymatic oxidation and bio-coloration/SEM image of swelled BC membranes

Phenolic compound/molar mass	Flavonoids representation
Catechol (110.1 g/mol)	
Catechin (290.26 g/mol)	
Hydroquinone (110.11 g/mol)	
Ferulic acid (194.18 g/mol)	
Swelled BC sample (SEM image obtained ×5000 magnification)	

## Materials and methods

### Materials and equipment

Commercially available BC gel from scooby tea fungus KOMBUCHA GET was obtained from Culver, CA, USA. Glucose and peptone were obtained from Merck Co. Ltd., Munchen, Germany while yeast extract was obtained from Sigma Chemical Co (St. Louis, Mo, USA). Analytical grade sodium hydroxide hydrate pellet and sodium chloride was purchased from Sigma Chemical Co (St. Louis, Mo, USA). Analytical grade glacial acetic acid and HPLC-grade methanol were purchased from Fisher chemical (Fair Lawn, NJ, USA). Sodium hydroxide (NaOH) from Sigma Chemical Co (St. Louis, Mo, USA). Commercial laccase (Novozym 51003) (EC 1.10.3.2) from *Myceliophthora thermophila* was obtained from Novozymes (Bagsveard, Denmark). The phenolic compounds namely catechol, catechin hydrate, hydroquinone, ferulic acid and quercetin dehydrate were purchased from Sigma Chemical Co (St. Louis, Mo, USA). Static cultivation of BC was conducted in an incubator (SI-600R, JEIO TECH Co., Daejeon, Korea). The swelling of BC was performed using an ultrasonic bath (VWR USC 200TH, VWR International, Kuala Lumpur, Malaysia). The internal morphology of BC samples was examined by scanning electron microscope (SEM, JSM-7600F, JEOL Korea Ltd., Seoul, Korea). All the polyphenols were characterized by ^1^H NMR spectroscopy, using a Bruker Avance III 400 (400 MHz). DMSO-d_6_ were used as deuterated solvents, using the peak solvent as internal reference. The color measurements were carried out using a spectrophotometer (illuminant D65 at 570 nm).

### Production of bacterial cellulose

The bacterial cellulose was produced using HS medium composed of: 20 g/L glucose, 5 g/L yeast extract, and 3 g/L peptone (Chen et al. 2015). Commercial BC gel from scoby was added to the culture medium in a liquor ratio of 1:5 (v/w). Static cultivation was conducted in the stationary phase at 26 °C for 8 days in an incubator (SI-600R, JEIO TECH Co., Daejeon, Korea). After cultivation, the BC was washed with distilled water at 100 °C for 5 min. Afterwards the BC was bleached with 10% (v/v) of H_2_O_2_ solution at 90 °C for 60 min in a water bath (110 rpm). For further studies of immobilization, BC samples were swelled with 8% of NaOH solution in a liquor ratio of 1:10 (v/w) for 30 min under ultrasounds. The ultrasonication was conducted in a sonicator bath at a fixed frequency 45 kHz and power intensity at 120 W. Afterward, the BC samples were dried for 2 h at 50 °C (Song et al. 2017).

### Laccase immobilization on bacterial cellulose membranes

BC samples were cut into small pieces (2 cm^2^) and immersed in 90U/mL of native laccase in acetate buffer (pH 4.0), the immobilization proceed at two different temperatures, room temperature and 4 °C, for 12 h.

### Measurement activity of immobilized laccase

The activity of laccase immobilized on BC was measured by incubating the BC-containing laccase samples with 3 mL of ABTS (2,2′-Azino-bis(3-ethylbenzothiazoline-6-sulfonic acid) (0.5 mM) at 50 °C for 60 min. Samples were taken every 10 min. and the absorbance was measured by UV–visible spectrophotometry at 420 nm. The activity of immobilized laccase was calculated using the following Eq. 1.

1 $${\text{Activity of immobilized laccase }}\left( {{\text{U/g}}_{\text{BC}} } \right)\,{ = }\,\frac{{{\text{k }} \times {\text{Vtotal }} \times 1 0^{ 6} }}{{{\text{M}}_{\text{BC}} \times \varepsilon }}$$

### Laccase-mediated polymerization with different monomers

For laccase-mediated polymerization experiments, four monomers namely catechol, catechin, ferulic acid and hydroquinone were selected. Each phenolic compound (10 mM) was dissolved in 100 mM sodium-acetate buffer, pH 5.0 using methanol. Than the BC samples, containing laccase previously immobilized at room temperature and at 4 °C, were immersed on 5 mL of this buffer and the polymerization was conducted overnight. Afterwards, the samples were washed to remove the unbound polymer.

### Laccase-mediated bio-coloration

Based on the polymerization studies described previously, native laccase immobilized at 4 °C was selected to catalyze the in situ polymerization of catechol and catechin hydrate for BC coloration. Each monomer was prepared in acetate buffer, pH = 5 at different concentrations, 1, 5, 10, 20 mM and placed in contact with the BC-containing laccase membranes. After overnight incubation the BC membranes were washed in 1:10 distilled water at room temperature for 50 min to eliminate the unbound polyphenol. The samples were posteriorly dry and the spectral values were determined.

### UV–Vis spectroscopy of polymeric solutions

The monitoring of UV–visible spectra was performed using UV–visible spectrophotometry in the wavelength range of 300–700 nm. The polymerization of the four monomers was followed by UV–visible spectroscopy using a 96-quartzo microplate reader (SynergyMx, Shimadzu, Japan). For this, the residual bath of the laccase-catalyzed solution is evaluated at wavelengths from 400 to 700 nm.

### NMR analysis of polymeric solutions

The ^1^H NMR spectroscopy of the new polyphenols was determined using a Bruker Avance III 400 (400 MHz). DMSO-d_6_ was used as deuterated solvent, using the peak solvent as internal reference.

### MALDI-TOF MS spectrometry

MASS SPECTRA analysis of the new oligomers was verified by Matrix-Assisted Laser Desorption/Ionization with time-of-flight (MALDI-TOF) using 2,5-dihydroxy benzoic acid (DHB) as the matrix (≥ 99.5%). The mass spectra were acquired on an Ultra-flex MALDI-TOF mass spectrophotometer (Bruker Daltonics GmbH) equipped with a 337 nm nitrogen laser. For this, the samples were dissolved in a TA30 (30% acetonitrile/70% TFA) solution and mixed with a 20 mg/mL solution of DHB (1:1). Then a volume of 2 μL was placed in the ground steel plate (Bruker part no. 209519) until dry. The mass spectra were acquired in analyzed using in linear positive mode.

### Determination of color strength

The color strength of the BC membranes after in situ polymerization was determined by K/S quantification obtained spectrophotometrically by a Datacolor apparatus at standard illuminant D65. The K/S values were calculated based on the Kubelka–Munk Eq. (2):

2 $$K/S = \frac{{\left( {1 - R} \right)^{2} }}{2R}$$

where *K* is the absorbance coefficient, *S* is the scattering coefficient, and *R* is the reflectance. The K/S value is directly proportional to the amount of color present on the surface of bacterial cellulose membranes and is calculated using the sum of reflectance values for all the wavelengths. The values were presented as checksum *K/S* (the sum of *K/S* of all wavelengths of the samples spectra).

### Color fastness test

The color resistance test of BC colored samples consisted on three sequential washing steps. Each step was performed at 25 °C for 120 min. at 100 rpm. After the last step, the BC samples were rinsed with tap water and dried overnight at 40 °C for color strength evaluation.

## Results

### Laccase immobilization onto BC membranes

Bacterial cellulose membranes were used as supports for laccase immobilization. Results of Table 2 show that laccase after immobilization, at room temperature and at 4 °C, retained up to 83 and 88% of its initial activity, respectively.

**Table 2:** Activity (U/g_BC_) and protein loading (mg/g_BC_) of laccase after immobilization (RT and 4 °C) onto BC membranes

	Activity (U/g_BC_)		Protein loading (mg_protein_/g_BC_)	
	RT^a^	4 °C	RT^a^	4 °C
Free laccase	7.2 ± 0.2	8.7 ± 0.3	–	–
Immobilized laccase	6.0 ± 0.5	7.7 ± 0.6	2.2 ± 0.3	2.7 ± 0.2

^a^Room temperature

### Laccase polymerization of phenolics by immobilized laccase

#### UV/visible spectra

**Fig. 1:**
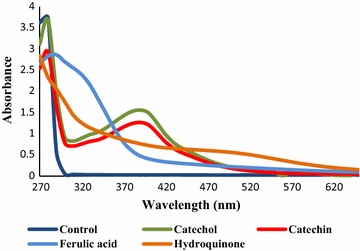
UV–visible spectra of treatment liquor solutions after polymerization by native laccase immobilized at 4 °C; the polymerization experiments were conducted at pH = 5, 50 °C, overnight

Four phenolic compounds belonging to the flavonoids family were oxidized by laccase, previously immobilized at room temperature and at 4 °C. Figure 1 shows the UV/visible spectra of the final solutions after enzymatic oxidation of the monomers. The results were more pronounced, in terms of color absorption and color depth, after oxidation with laccase immobilized at 4 °C. For this reason we present herein only the results obtained with enzyme immobilized at 4 °C.

The buffer solution (control) and the solutions of each polymer were all clear and colourless in the first hours of incubation. After addition of the phenolic solutions to BC samples containing laccase previously immobilized, they produce deep coloration, resulting in higher absorption in the visible region of the spectra (Fig. 1).

#### ^1^H NMR analysis of the new oligomers

**Fig. 2:**
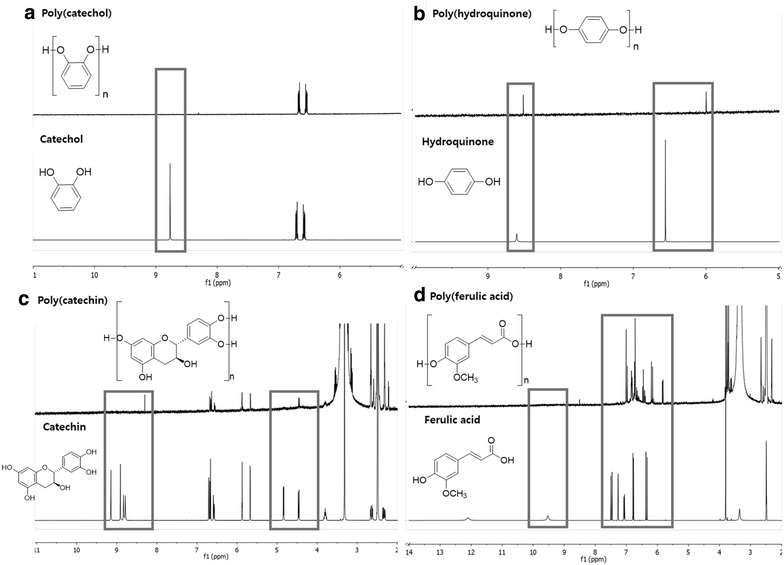
^1^H NMR spectra of **a** poly(catechol), **b** poly(hydroquinone), **c** poly(catechin) and **d** poly(ferulic acid); (^1^H NMR were acquired at 400 MHz, using deuterated dimethylsulfoxide, 298 K); the polymers were obtained after polymerization with laccase immobilized at 4 °C at pH = 5, overnight

The final solutions were analyzed in order to predicte the oligomers formed during flavonoids oxidation. Figure 2 presents the ^1^H NMR spectra of all the oligomers produced. Figure 2a reveals that the aromatic peaks of catechol, that appear in the monomer between δ_H_ 6.69–6.72 and 6.57–6.60 ppm, suffer a slightly shift appearing between δ_H_ 6.64–6.70 and 6.5–6.575 ppm in the oligomers. The more significant change, is the disappearance of the OH signal that appears at δ_H_ 8.75 ppm in the monomer, confirming that the polymer was formed, by oxygen-bond linkage. Only one type of polymer is detected by ^1^H NMR, confirming that the immobilized laccase has selectivity for the formation of homogenous catechol polymer (as depicted in spectra 2a).

The same behaviour occurred for hydroquinone spectra (Fig. 2b), which is composed by two peaks: the aromatic protons at δ_H_ 6.56 ppm, and the OH at δ_H_ 8.60 ppm. The polymerization is detected by the disappearance of the OH proton signals in the starting material, and a significant chemical shift of the aromatic protons to δ_H_ 5.99 ppm. In catechin ^1^H NMR, the phenolic OH protons appeared between δ_H_ 8.79–9.15 ppm, all as singlets. In the poly(catechin) spectra (Fig. 2c), the OH protons disappeared. The remained peaks, are kept in the same chemical shift, which lead us to assume that the polymerization occurred via oxygen-linkage. Analysing the ^1^H NMR of ferulic acid and its polymeric derivatives (Fig. 2d), is indistinguishable the presence of the carboxylic acid proton at δ_H_ 12.10 ppm, and the aromatic OH at δ_H_ 9.53 ppm in the starting material. The aromatic and allyl protons are situated between δ_H_ 6.33 and 7.50 ppm. The spectra of poly(ferulic acid) showed very differences concerning the pattern of the protons, which proved the polymerization.

#### MALDI-TOF analysis

**Fig. 3:**
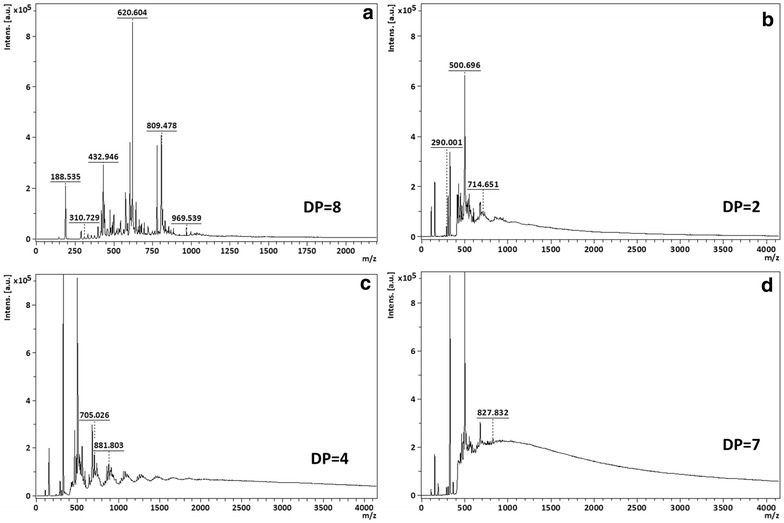
MALDI-TOF analysis of polymers obtained after polymerization with laccase immobilized at 4 °C; the polymerization was conducted at 50 °C, overnight at pH = 5: **a** poly(catechol; **b** poly(catechin); **c** poly(ferulic acid); **d** poly(hydroquinone)

The new oligomers produced were also analyzed by MALDI-TOF spectrometry in order to infer the degree of oligomerization (Fig. 3). As it can be seen in the spectra, polymerization of all phenolics resulted in the formation of oligomers with different size (Table 3). The MALDI-TOF spectra obtained show that the samples are very heterogeneous making hard to define the polymerization pattern for all the oligomers produced. This heterogeneity might be related with the different oligomer size obtained and the ionization events occurred during the analysis. The data obtained is although valuable to confirm that polymerization really occurred and to gather the medium degree of oligomerization for each polymer obtained (see Fig. 3). From data analysis it is possible to observe that oxidation of catechol and hydroquinone give rise to longer polymers, while smaller oligomers are obtained after oxidation of catechin and ferulic acid.

**Table 3:**
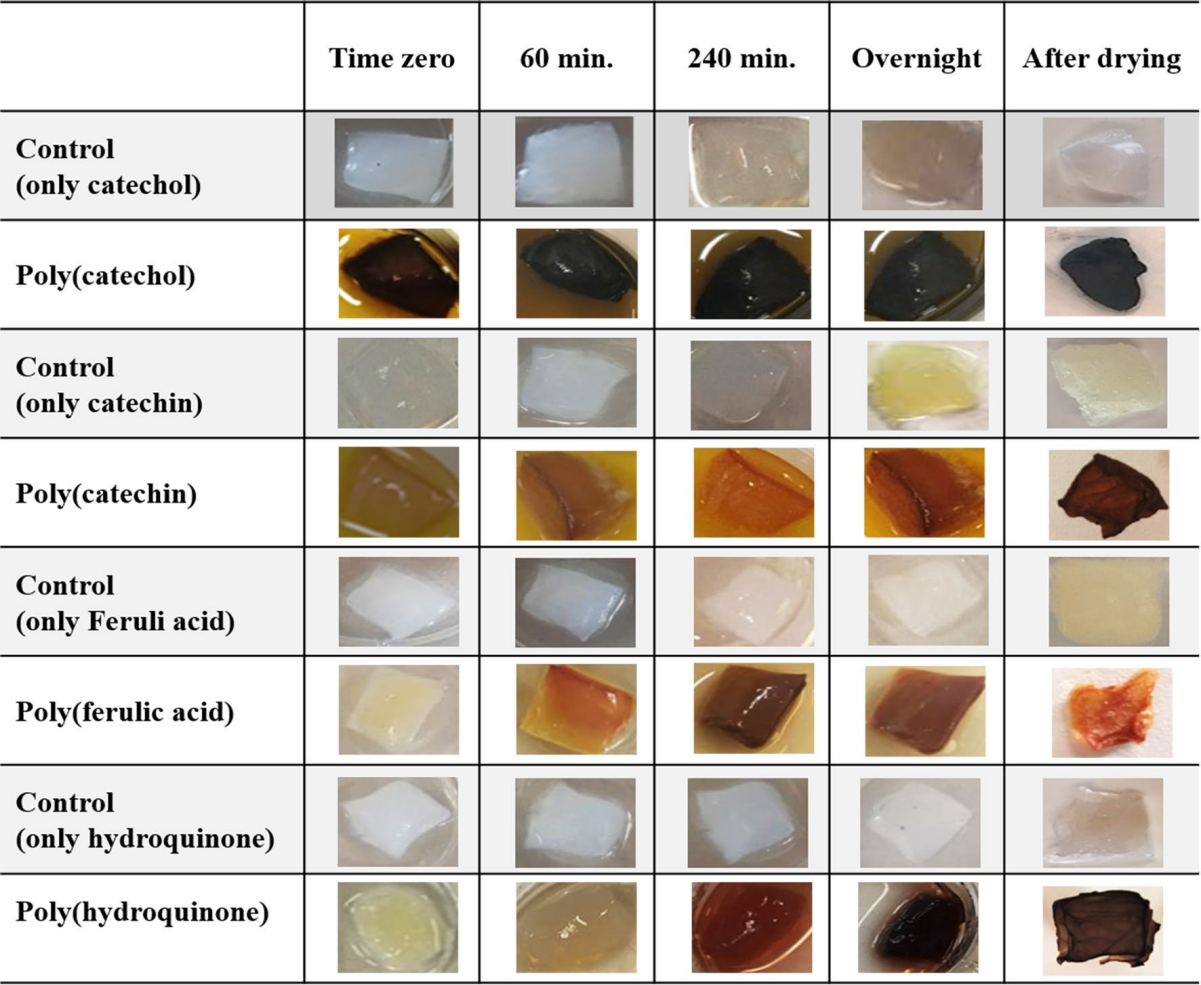
Bacterial cellulose samples after polymerization of catechol, catechin, ferulic acid and hydroquinone with native laccase immobilized at 4 °C; the polymerization was conducted at 50 °C overnight

#### In situ polymerization–visual evaluation

The in situ polymerization of the flavonoids by immobilized laccase give rise to strong of the BC supports. The flavonoids oxidation was responsible for the color obtained and increases with the incubation time, reaching very strong depth after 240 min. The samples were kept for overnight incubation to estimate if longer incubation periods would improve significantly the coloration. From the results obtained this was valid for both catechin and hydroquinone. The highest coloration with poly(catechol) was achieved after 60 min and ferulic acid seemed to lose color when incubation time increased to overnight. It is noteworthy after visual evaluation of colored samples the great potentiality of the bacterial cellulose membranes to be biocolorized (Table 3).

#### Laccase-assisted bio-coloration of BC membranes by immobilized laccase

##### UV/Visible characterization

**Fig. 4:**
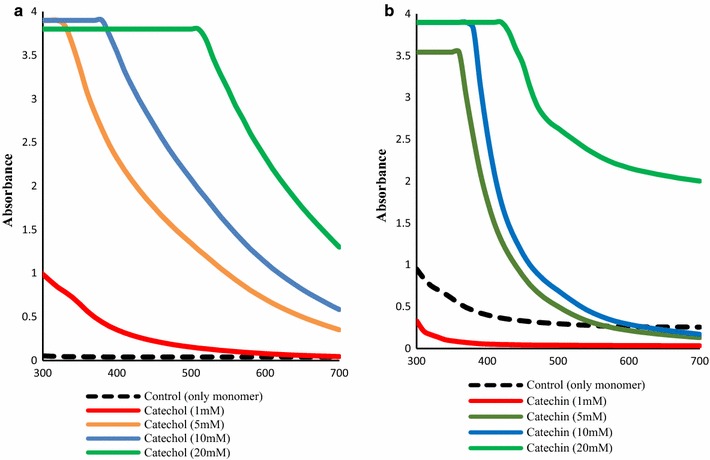
UV–Vis spectra of the solutions before and after polymerization with laccase at 50 °C overnight of **a** catechol and **b** catechin hydrate

Taking into consideration the role of BC samples as enzyme support for phenolics polymerization, we proceed with the bio-coloration studies. For this, different concentrations of both monomers, catechol and catechin, were studied for the enzymatic oxidation by immobilized laccase. The products of this oxidation would confer color to the support. As it can be depicted from the analysis of Fig. 4, the addition of both monomers to the BC samples containing immobilized laccase lead to a significant increase of the absorption in the visible region. The increase is directly proportional to the concentration of monomer used.

##### Color strength of BC samples after enzymatic coloration

Table 4 shows the *K/S* values of BC samples after bio-coloration with catechol and catechin oxidized by immobilized laccase. The sum of *K/S* of control samples (only laccase and only monomers) is low, corresponding to the natural coloration given by the enzyme and the monomer without oxidation. The checksum *K/S* values increase with the concentration of monomer used, as previously confirmed by UV/visible spectra, giving yellow and dark brown color to the BC samples.

**Table 4:**
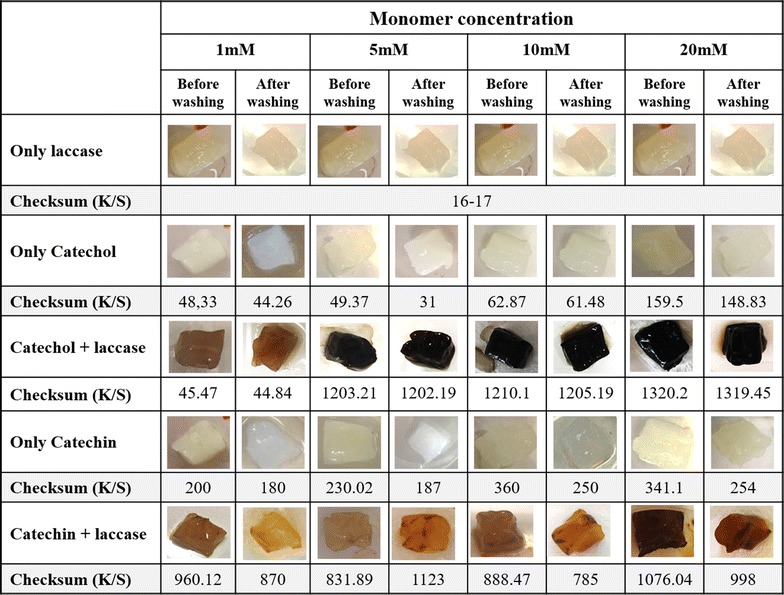
Bacterial cellulose samples before and after in situ polymerization of catechol and catechin with immobilized laccase at 50 °C, overnight; spectra values are presented as the sum of *K/S* values

##### Color fastness to washing

**Fig. 5:**
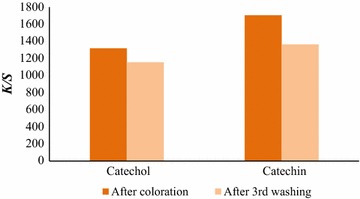
Color strength of BC samples after bio-coloration with poly(catechol) and poly(catechin) and after three cycles of washing (each cycle: water at 25 °C, 120 min., 100 rpm)

In this work, the BC samples colorized by the polyphenolics (catechol and catechin) were subjected to three cycles of washing to evaluate the color resistance on these specific porous materials (Fig. 5). From the data obtained it is possible to detect color reduction of samples colorized by both polymers, being this reduction more pronounced on samples containing poly(catechin). This is also perceptible by visualization of the colored samples which present low color homogeneity and therefore more propensity to be washed-out.

## Discussion

In a previous study we have evaluated the best conditions for the immobilization of laccase onto bacterial cellulose membranes (Song et al. 2017). Considering the best operational immobilization conditions, the goal herein was to immobilize native laccase onto bacterial cellulose membranes maintaining its catalytic activity for posterior catalysis. The results obtained are in accordance with the data from literature in which high levels of activity are reported after laccase immobilization (Frazão et al. 2014). The discrepancy observed between protein loading and the enzyme activity values are related with the steric hindrance of the adsorbed enzyme and mass transfer resistance. After immobilization, the media is changed from water to porous BC, reducing mass transfer and the binding efficiency between the enzyme and the substrate. In our study, and as we discuss further, the activity measured in terms of polymer formed, was not influenced by this activity reduction. Since laccase immobilized at 4 °C retained the highest activity values, these samples were used for further experiments.

Proceeding with the first experiments of oxidation of the four flavonoids we observed that the results were more pronounced, in terms of UV/Visible absorption and color depth, after oxidation with laccase immobilized at 4 °C. After addition of the phenolic solutions to BC samples containing laccase previously immobilized, they produce deep coloration, resulting in higher absorption in the visible region of the spectra (Fig. 1). This increase of color absorption is related with the amount of oligomers formed. The oligomers production is based on mechanisms proposed and well described in literature which reveal that laccase-catalyzed polymerization might proceed mainly through the nucleophilic attack rather that radical mechanism (Osman et al. 2007; Sun et al. 2013).

The new polymers were characterized by ^1^H NMR and MALDI-TOF spectrometry and all spectra obtained confirmed the polymerization of all monomers. Moreover, only one type of polymer is detected by ^1^H NMR, confirming that the immobilized laccase has selectivity for the formation of homogenous polymers. After MALDI-TOF analysis of the new polymers it was possible to infer that the oxidation with immobilized laccase gave rise to different polymerization degrees depending on the monomer. Depending on their class, flavonoids differ on the level of oxidation, even under the same experimental conditions (Kumar and Pandey 2013; Pourcel et al. 2007). The enzymatic oxidation of catechol and hydroquinone produced bigger oligomers while the oxidation of catechin and ferulic acid gave rise to smaller oligomers.

The in situ polymerization of the flavonoids by immobilized laccase induced strong coloration to the BC supports. The flavonoids oxidation was responsible for the color obtained which increases with the incubation time, reaching very strong depth after 240 min. It is noteworthy after visual evaluation of colored samples, the great potentiality of the bacterial cellulose membranes to be bio-colorized. Their high porosity allows that a higher amount of polymer remain inside the material in contrast to other materials like cotton, to which the new polymers do not have significant affinity (Calafell et al. 2007; Song et al. 2017). The color strength, visually evaluated (Table 3), corroborates the MALDI-TOF results obtained. Higher degrees of polymerization correspond to higher coloration levels of BC membranes. Catechol and hydroquinone presented higher oligomerization degree and gave rise to a dark brown coloration of BC samples. On the other hand, the small oligomers obtained after enzymatic oxidation of catechin and ferulic acid, are responsible to impart yellow-orange coloration to the substrates.

At this stage, it is possible to observe that some polymer is still present on the solution and/or maybe wash-out from the surface of the BC samples. To optimize the oxidation of the phenolics and evaluate the color resistance, we further performed the polymerization studies with catechol and catechin.

Taking into consideration the role of BC samples as enzyme support for phenolics polymerization, we proceed with the bio-coloration studies. The addition of both monomers to the BC samples containing immobilized laccase lead to a significant increase of the absorption in the visible region. This increase is directly proportional to the concentration of monomer used. The spectral values obtained after monomers oxidation reveal that the checksum *K/S* values increase with the concentration of monomer used, as previously confirmed by UV/Visible spectra, giving yellow and dark brown color to the BC samples. These results encourage us to predict that depending on the final intended, it is possible to achieve a pallet of colors that covers the yellow, the orange, the light and the dark brown. The color depth can also be tuned by variation of the initial monomers concentration.

After coloration, the unbound material was removed by washing of the samples with water at 60 °C followed by color strength evaluation. The sum of *K/S* values decreased after washing in a higher extent on the samples colorized with poly(catechin). The samples colorized by poly(catechol) showed a lower *K/S* loss after washing. This is a surprising behavior, since other reports presented high color reduction after only one washing (Bai et al. 2016; Kim et al. 2008). The high porosity of the BC material might be responsible for the higher polymers entrapment and higher color resistance.

The enzymatic coloration of textiles with compounds produced enzymatically by laccase have usually low affinity towards the fibres, demonstrating no covalent fixation (2007). The color fastness of BC samples after coloration was evaluated and color reduction was detected for by both polymers, being more pronounced on samples containing poly(catechin). This is also perceptible by visualization of the colored samples which present low color homogeneity and therefore more propensity to be washed-out.

Despite the mechanism of dyestuff fixation onto cellulosic materials has not been clarified, we might speculate that the high porosity of the BC membranes and the lower solubility of the oligomers produced, can be the key factors for the success of the coloration. The low soluble oligomers can go deeply into the highly porous material remaining stable when the washing process is applied. Higher oligomers are thus more difficult to remove by washing, in the case of poly(catechol) than smaller catechin oligomers.

Flavonoid compounds like catechol, catechin, ferulic acid and hydroquinone can polymerize and develop deep colors by laccase action. Considering this, we have described a process for the bio-coloration of bacterial cellulose membranes via phenolics oxidation by immobilized laccase. The immobilization of laccase onto bacterial cellulose supports has been exploited already by several researchers but, as far as we know, no practical applications have been developed.

Herein, the catalyst was efficiently immobilized onto the porous BC membranes maintaining up to 88% of its activity. The BC membranes were then successfully colorized by oxidation of catechol and catechin, catalyzed by laccase previously immobilized onto these supports. The color depth may vary depending on the initial amount of monomer being the highest level obtained with poly(catechol) coloration. The highest color depth and level of fixation obtained for catechol are related with the longer oligomers produced during enzymatic oxidation, which confer stronger coloration to the materials surface.

## Abbreviations

BC: bacterial cellulose
UV/Vis: ultra-violet/visible
DMSO: dimethyl sulfoxide
